# Effectiveness study of recrystallisation method in pharmaceutical salt production from processed salt with zero waste concept

**DOI:** 10.1016/j.heliyon.2024.e30472

**Published:** 2024-04-29

**Authors:** T. Widjaja, A. Altway, S. Nurkhamidah, Y. Rahmawati, W. Meka, A. Alifatul, D. Hartanto, R. Sari

**Affiliations:** a Chemical Engineering Department, Institut Teknologi Sepuluh Nopember, Sukolilo, Surabaya, 60111, Indonesia; b Chemical Department, Institut Teknologi Sepuluh Nopember, Sukolilo, Surabaya, 60111, Indonesia; c Pharmaceutics Department, Universitas Airlangga, Tambaksari, Surabaya, 60132, Indonesia

**Keywords:** Impurities, Pharmaceutical salt, Precipitating agent, Recrystallisation, Salt

## Abstract

Indonesia's vast archipelago offers abundant seawater resources, holding the potential for salt production. Salt, a vital commodity in human life, typically contains sodium chloride and impurities like Ca^2+^, Mg^2+^, SO_4_^2−^, and K^+^. Pharmaceutical salt is an industrial category adhering to pharmacopoeial standards regarding sodium chloride levels and impurity content, ensuring quality for drug preparations in Indonesia. Prior research indicates that recrystallisation, specifically evaporation crystallisation, enhances salt quality by increasing NaCl content. Chemical precipitating agents like NaOH and Na_2_CO_3_ can be introduced to improve salt purity further. This study aims to identify optimal conditions for pharmaceutical salt production from processed salt raw materials, considering crystallisation time, stirring speed, chemical additives (NaOH and Na_2_CO_3_), and double crystallisation stages. The method commences with pre-treatment, involving salt dissolution in distilled water to saturation, with the addition of precipitating agents as per designated variables. Precipitates formed from precipitating agents (NaOH and Na_2_CO_3_) are isolated through filtration. The filtrate undergoes evaporation crystallisation at 103 °C, varying between single and double crystallisation. Salt crystals are separated, dried, and weighed to calculate yield. Pharmaceutical salt is analysed for water content, NaCl, and impurities (Ca^2+^, Mg^2+^, SO_4_^2−^, and K^+^). The optimal conditions for pharmaceutical salt production were double crystallisation with a 20 % excess of chemicals (NaOH and Na_2_CO_3_), 100 min of crystallisation time, and a stirring speed of 600 rpm. This yielded a 15 % NaCl content of 99.87 %, Mg^2+^ at 0 ppm, Ca^2+^ at 69.6 ppm, SO_4_^2−^ at 366 ppm, K^+^ at 370 ppm, and water content at 0.166 %. Notably, the pharmaceutical salt production process generates no waste, as byproducts like Mg(OH)_2_ and CaCO_3_ can be recycled and hold commercial value. However, it is essential to re-evaluate raw materials and technologies to address the market's high cost and competitiveness issues.

## 1 Introduction

Indonesia is the world's largest archipelagic nation. In addition to having extensive marine waters that cover 71 % of its total area, this geographical condition has resulted in a coastline that stretches for approximately 95,181 km, ranking it as the second-longest coastline in the world, reported by Ref. [1]. This presents a significant opportunity for Indonesia to harness its marine resources, particularly in producing economically valuable salt. Salt is an essential commodity in human life. Typically, salt is produced from raw materials such as seawater due to its high salt content.

Additionally, salt can be obtained from rock salt mining and saltwater wells (brine) reported by Tansil et al., 2016. Salt is commonly found in white crystals and primarily consists of sodium chloride (NaCl) and impurities such as Ca^2+^, Mg^2+^, SO_4_^2−^, and K^+^, among others. Through acid-base reactions, salt is formed from cations (positive ions) and anions (negative ions), resulting in a neutral compound. Functionally, salt can be categorised as either consumer salt or industrial salt. Consumer salt serves household needs and fulfils electrolyte requirements in the human body. On the other hand, industrial salt finds applications in various industries, including chemical, food, pharmaceutical, and more, as reported by Geertman, 2000 [2].

Pharmaceutical salt, categorised as industrial salt, is typically sodium chloride that complies with pharmacopeial standards, specifications and guidelines for drug formulations in Indonesia. According to prevailing standards, pharmaceutical salt should contain sodium chloride with a purity of >99 % and minimal impurities. The maximum allowable levels for impurities include Ca^2+^ and Mg^2+^ at 100 ppm, SO_4_^2−^ at 200 ppm, and K^+^ at 500 ppm, and it should be free of heavy metals, as reported by Geankoplis, 2003. Pharmaceutical salt is used as a raw material in producing various pharmaceutical products such as infusion solutions, cosmetics, medicines (tablets and syrups), vaccine solvents, and even blood cleansing solutions.

Despite Indonesia's geographic potential for salt production, the reality is that the country still imports industrial salt. The high volume of salt imports in Indonesia is attributed to the quality of salt produced by local salt farmers, which needs to meet standards. The average NaCl content in locally produced salt ranges from 93 % to 97 %, with impurities exceeding acceptable limits.

One of PT Garam's commercial salt products is refined salt. Refined salt is processed from raw materials such as coarse salt or solar salt, resulting in non-iodised acceptable salt meeting the standards for consumer salt according to SNI 3556 in 2016. The composition of refined salt includes a sodium chloride content of 95.5 %, Ca^2+^ and Mg^2+^ at 5100 ppm, K^+^ at 2200 ppm, and SO_4_^2−^ at 18800 ppm. However, these salt compositions still need to meet the requirements for pharmaceutical salt as per pharmacopoeia standards. Additionally, Presidential Regulation PP No. 126/022 stipulates that by 2024, Indonesia's national salt needs must be met by domestic producers, with imports prohibited. This poses a challenge for salt production in Indonesia, where most salt farmers continue to use conventional methods, primarily open evaporation crystallisation, influenced by weather conditions, and have yet to produce salt meeting established standards. This challenge is compounded by the increasing demand for salt each year. Therefore, there is a need for innovative salt processing methods to produce pharmaceutical salt, enhance the competitiveness of local salt against imported salt, and prevent future salt crises, particularly for pharmaceutical needs.

Previous research has proposed several methods to improve the quality of salt produced by local salt farmers for industrial use. Recrystallisation is a technique for purifying solid substances from a mixture by redissolving the mixture in a suitable solvent and then crystallising it again. Based on research by Ref. [3], recrystallisation has been shown to improve the quality of salt in terms of its NaCl content, increasing it from an initial content of 80–85 % to 94–98 %. According to the [4] findings, recrystallisation is suitable for pharmaceutical salt production based on various case studies in Europe, Asia, and Africa. The recrystallisation process can automatically produce pharmaceutical salt without any additives. In the production of pharmaceutical salt, if the raw material contains impurities such as high potassium or sulfate levels or if the impurity content in the pharmaceutical salt product is exceptionally high, as is the case in China's standards, a double crystallisation or recrystallisation process can be performed to lower the impurity content reported by Ref. [5]. Recrystallisation is recommended for pharmaceutical salt synthesis due to its simplicity, ability to produce high-purity pharmaceutical salt, cost-effectiveness, and ease of process control, as reported by Ref. [6].

Another method to enhance the purity of salt is by adding chemicals that function as precipitating agents, and the waste becomes a product that can be utilised; it is called the zero waste concept. A solution supplemented with a precipitating agent will form solid particles known as precipitates. Based on research conducted by Ref. [7], adding Na_2_CO_3_ in salt purification can reduce impurity content in the salt, specifically reducing calcium (Ca^2+^) content. On the other hand, according to a study by Ref. [8], adding a chemical like NaOH can increase the NaCl content in salt from 92 % to 96 %. This is because the OH- ions in NaOH bind to impurities such as Mg^2+^, while Na^+^ ions combine with Cl^−^ ions, increasing NaCl content.

Therefore, this research uses the recrystallisation method to address the competitiveness issues between local and imported salt in producing pharmaceutical-grade salt by pharmacopeial standards. The method involves several stages, starting with the pre-treatment phase, where raw salt is dissolved in water until saturation and a precipitating agent is added based on predetermined variables. Subsequently, the crystallisation stage is carried out, considering variables such as crystallisation time, stirring speed, and the number of crystallisations, including both single and double crystallisations. The treatment of single and double recrystallisation aims to determine the influence of the extent of crystallisation on the quality of the produced salt. The double recrystallisation method involves recrystallising the processed salt in two stages, where, following the addition of precipitating agent (NaOH and Na_2_CO_3_), the salt solution is recrystallised, separated from the mother liquor, and then redissolved for a second recrystallisation process. This results in the production of pharmaceutical-grade salt with high purity and low impurities levels.

## 2 Materials and methods

### 2.1 Materials

The production of pharmaceutical-grade salt utilises primarily raw salt obtained from PT Garam, a non-iodised acceptable salt. Other materials used include NaOH, Na_2_CO_3_, and distilled water.

### 2.2 Pre-treatment stage

This stage involves dissolving the raw salt and filtering out impurities such as sand, gravel, and soil, followed by adding precipitating agents in the form of chemicals, namely NaOH and Na_2_CO_3_. Subsequently, precipitation and sediment filtration are carried out. Adding NaOH and Na_2_CO_3_ chemicals serves the purpose of binding impurities, specifically metal ions like magnesium and calcium. The salt dissolution process takes 20 min, while the chemical addition process lasts 30 min. Both processes occur at a temperature of 30 °C, a pressure of 1 atm, and with a stirring speed of 400 rpm.

### 2.3 Crystallisation stage

In this stage, the salt solution that has undergone the pre-treatment stage will be crystallised by heating it on a hotplate at 103 °C. The crystallisation process is carried out either as a single or double crystallisation. In the case of double crystallisation, the salt obtained from the first crystallisation, which has already been filtered, is dissolved again in distilled water and then crystallised by heating on a hotplate. The crystallisation was conducted by varying the crystallisation time and stirring speed, which had been predetermined. The filtered product was then placed in the oven at 60 °C for 24 h.

## 3 Results and discussion

### 3.1 Yield of pharmaceutical salt

**Fig. 1:**
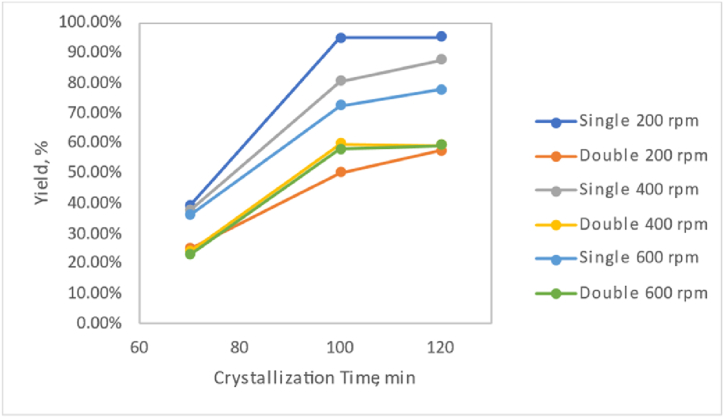
Relationship graph of pharmaceutical salt yield with crystallisation time in the variable without chemical addition.

Based on Fig. 1, it can be observed that the yield value at a crystallisation time of 120 min is the highest compared to crystallisation times of 100 min and 70 min. The yield increases with increasing crystallisation time. Yield is obtained by dividing the mass of the product by the mass of the reactant or raw material. This is consistent with the research by Rositawati et al., 2013, which suggests that as the crystallisation time increases, the quantity of salt crystals produced also increases. The increase in salt mass formed with longer crystallisation times is due to more solvent (water) evaporating over time, forming more salt crystals.

**Fig. 2:**
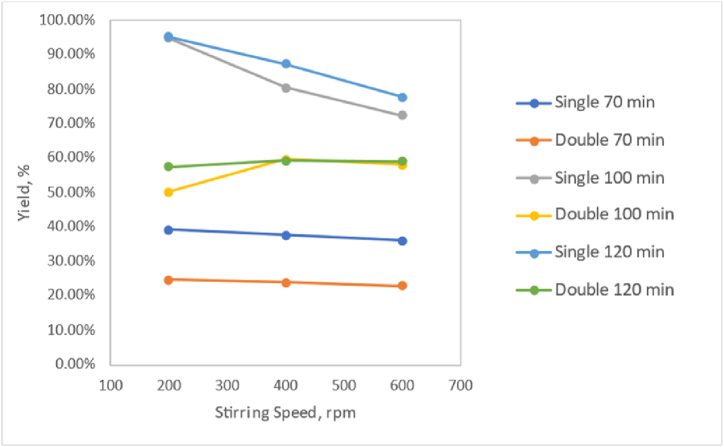
Relationship graph of pharmaceutical salt yield with stirring speed in the variable without chemical addition.

According to the graph in Fig. 2, for all crystallisation times, namely 70, 100, and 120 min, the yield at a stirring speed of 200 rpm is higher than at 400 rpm or 600 rpm. However, during double crystallisation at 100 min and 120 min, the yield does not consistently increase with decreasing stirring speed. Higher stirring speeds generally tend to reduce the mass of the pharmaceutical salt formed. According to a report by Ref. [6], stirring affects the crystal formation process after nucleation, where stirring helps crystal nuclei collide with each other or with the beaker glass walls, allowing them to bind to other crystal nuclei. According to a report by Ref. [9], stirring affects the crystals formed during crystallisation. Higher stirring speeds during crystallisation accelerate the nucleation phase. However, higher stirring speeds result in a slower growth rate during crystal growth. Lower stirring speeds produce larger crystals, which are easier to filter than smaller ones. According to a report by Ref. [6], the highest salt yield is achieved at the lowest stirring speed. However, optimising stirring speed does not significantly affect the yield of pharmaceutical salt formed. This aligns with the findings of this study, as different stirring speeds do not significantly impact the yield of pharmaceutical salt, and the highest yield is obtained at the lowest stirring speed of 200 rpm.

**Fig. 3:**
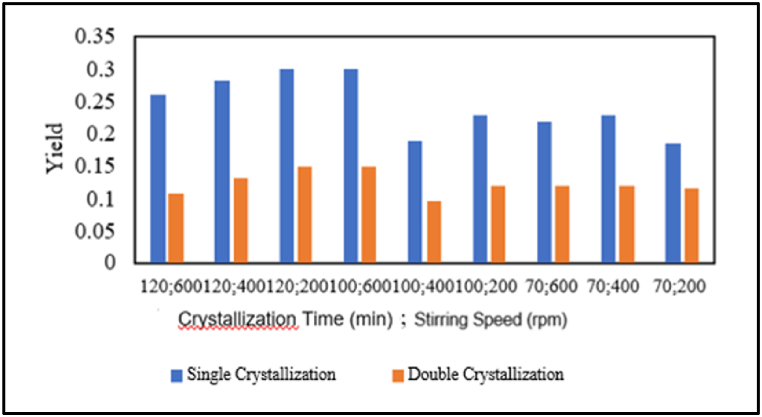
Effect of chemical addition on the mass of formed salt.

Based on Fig. 3, it can be observed that the addition of chemicals reduces the mass of pharmaceutical salt formed. This is because a significant portion of the mass that is lost does not participate in crystal formation and consists of impurities such as Mg and Ca. NaOH successfully binds impurities of magnesium, while NaOH and Na_2_CO_3_ bind calcium. This aligns with the research reported by Ref. [10], which suggests that NaOH and Na_2_CO_3_, as precipitating agents, bind impurities of Mg and Ca present in the salt solution, resulting in less mass of formed salt. This also corresponds with the findings reported by Ref. [11], indicating that the preparation or addition of NaOH and Na_2_CO_3_ chemicals results in fewer mass crystals than crystals formed without preparation or without the addition of NaOH and Na_2_CO_3_ chemicals.

**Fig. 4:**
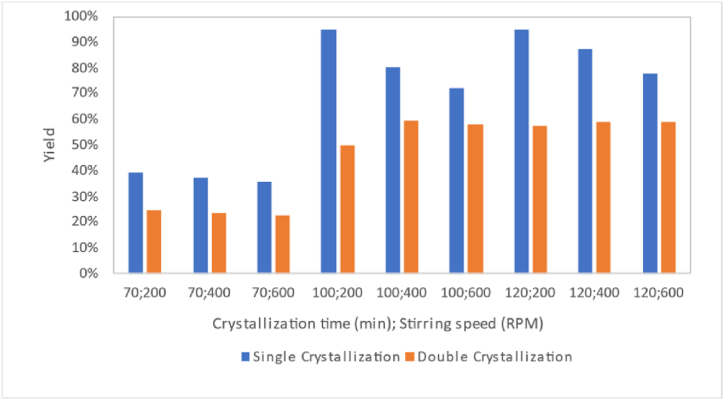
Effect of the number of crystallizations on pharmaceutical salt yield.

Based on the graphs in Fig. 4, it can be concluded that the yield of pharmaceutical salt decreases as the number of crystallisations increases from single to double crystallisation. This applies to all variables, including variables with or without pre-treatment and chemical addition. This is because, in double crystallisation treatment, two crystallisations occur, resulting in more NaCl and impurities dissolved in the mother liquor compared to a single crystallisation reported by Ref. [4].

### 3.2 Test results for pharmaceutical salt levels

The research results of the pharmaceutical salt production process from raw salt are not only based on mass and yield but also assessed for moisture content, NaCl, Ca^2+^, Mg^2+^, K^+^, and SO_4_^2−^.

#### 3.2.1 Water content

Concerning the moisture content test results in pharmaceutical salt, it can be concluded that the production of pharmaceutical salt using the recrystallisation method and the procedures performed can reduce the water content from 1.1 % to an average of 0.305 %. This is by the applicable pharmacopoeia standards, which specify a water content below 0.5 %. However, some pharmaceutical salts have a moisture content higher than 0.5 %. This may be due to prolonged storage, as the salt in this run was made one month before testing. The moisture content in salt is influenced by the operating conditions during the drying process and the hygroscopic nature of the salt, as reported by Ref. [1]. The drying process for salt products in this method is carried out in an oven for 24 h at a temperature of 60 °C for all variables. Additionally, the hygroscopic nature of salt, meaning its ability to absorb moisture, can lead to increased moisture content in the salt. Furthermore, excessive moisture content in pharmaceutical salt can promote bacterial growth and compromise sterility, as reported by Ref. [12].

#### 3.2.2 NaCl content

The recrystallisation treatment with various variables has successfully increased the initial NaCl content from raw salt material, ranging from 95.5 % to 96–99.8 %. According to pharmaceutical salt standards [13], the NaCl content should be >99 %. Based on the data, it can be concluded that the results meeting this standard are achieved in the 10th run with single crystallisation, where an excess of 20 % chemicals is added, with a crystallisation time of 100 min and a stirring speed of 400 rpm, and in the 13th run with double crystallisation, with an excess of 20 % chemicals, a crystallisation time of 100 min, and a stirring speed of 600 rpm. The effect of the crystallisation time on the NaCl content is presented in the graph below.

**Fig. 5:**
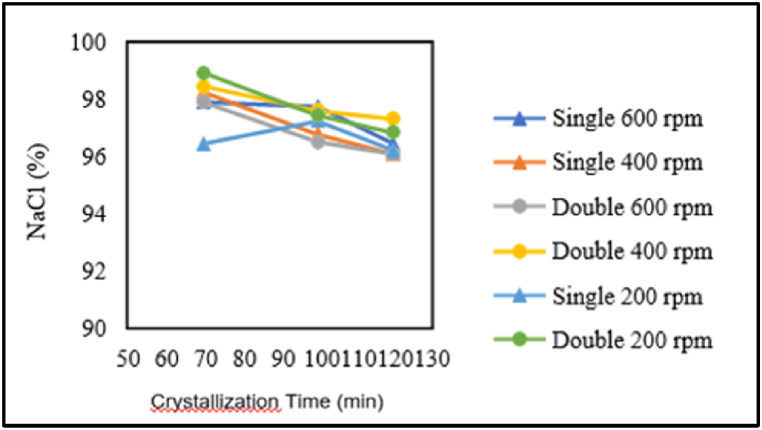
Relationship between crystallisation time and NaCl content.

In Fig. 5, for the pre-treatment without chemical additions, it can be seen that for single crystallisation, both at stirring speeds of 400 rpm and 600 rpm, and for double crystallisation, at stirring speeds of 200 rpm, 400 rpm, and 600 rpm, the highest NaCl content is achieved at a crystallisation time of 70 min, then decreases at 100 min, and finally decreases further at 120 min. Therefore, longer crystallisation times lead to a decrease in NaCl content. In contrast, for single crystallisation at a stirring speed of 200 rpm, the NaCl content increases when the crystallisation time is increased to 100 min. The NaCl content decreases again when the crystallisation time is extended to 120 min. For the pre-treatment with chemical additions, it can be observed that for single crystallisation, both at stirring speeds of 400 rpm and 600 rpm, and for double crystallisation at a stirring speed of 600 rpm, the longer the crystallisation time, the higher the NaCl content. However, the NaCl content decreases with longer crystallisation times for double crystallisation at a stirring speed of 400 rpm. In the pre-treatment without chemical additions, longer crystallisation times tend to decrease the NaCl content. This is consistent with the research conducted by Ref. [14], which showed that NaCl content decreases as the Ca^2+^ and Mg^2+^ impurity levels increase in the solution during prolonged crystallisation. This is because the crystallisation is done in batches, so the longer the crystallisation time, the more impurities get trapped in the NaCl crystals.

The influence of stirring speed on the NaCl content is presented in the graph below.

**Fig. 6:**
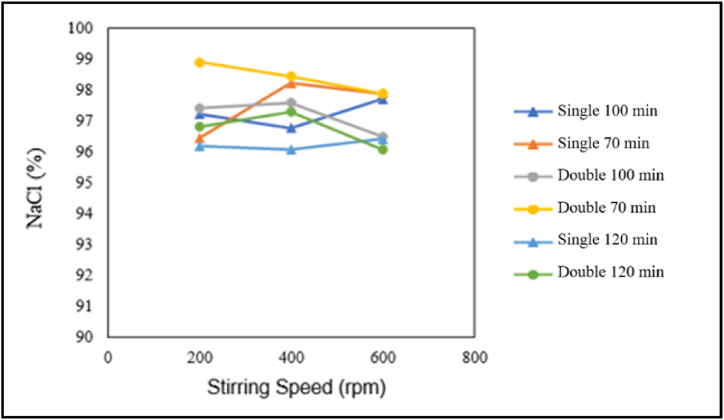
Relationship between stirring speed and NaCl content.

In Fig. 6, for the pre-treatment without chemical additions, it can be observed that for single crystallisation at a crystallisation time of 70 min and double crystallisation at crystallisation times of 100 min and 120 min, the highest NaCl content is obtained at a stirring speed of 400 rpm, and the NaCl content decreases at stirring speeds of 200 rpm and 600 rpm. On the other hand, for single crystallisation at crystallisation times of 100 min and 120 min, the highest NaCl content is achieved at a stirring speed of 600 rpm, then decreases at a stirring speed of 200 rpm, and the lowest NaCl content is obtained at a stirring speed of 400 rpm. Conversely, in the case of double crystallisation at a crystallisation time of 70 min, the highest NaCl content is obtained at a stirring speed of 200 rpm, and the NaCl content decreases as the stirring speed increases.

In conclusion, higher stirring speeds increase the NaCl content in the treatment without chemical additions. The increase in NaCl content with increasing stirring speed is due to the reduced porosity of the salt crystals, which leads to fewer crystals absorbing mother liquor-containing impurities, resulting in a decrease in NaCl content. This is consistent with the research by Ref. [15], which suggests that higher stirring speeds result in smaller and denser particle bonds. This is also supported by the study by Ref. [16], which states that higher porosity values lead to higher fluid absorption or more fluid sticking to the crystals. On the other hand, higher stirring speeds tend to decrease the NaCl content in the double crystallisation. In this case, the NaCl content is influenced more by the amount of solvent evaporated than by the size of the porosity. Higher stirring speeds increase the amount of solvent evaporated, leading to a higher concentration of impurities. This is supported by the research by Ref. [17], which suggests that higher stirring rates lead to higher evaporation rates. This statement is also supported by the research by Ref. [14], which states that the more solvent is evaporated, the more concentrated the impurities become, trapping more impurities on the salt crystals and causing a decrease in NaCl content. The uncertainty of the stirring speed's effect on NaCl content can be summarised as follows: stirring speed does not significantly impact NaCl content. This is consistent with the research by Ref. [6]. This suggests that stirring speed does not significantly influence NaCl content.

The influence of the number of crystallisations and the effect of chemical addition on NaCl content is presented in the graph below.

**Fig. 7:**
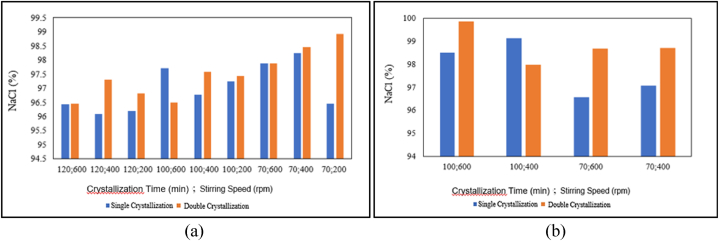
Relationship between number of crystallizations and NaCl content: (a) Without chemical additions, (b) with chemical additions.

Based on Fig. 7 (a), for the pre-treatment without chemical additions, it can be seen that double crystallisation tends to increase the NaCl content, except for a crystallisation time of 100 min at a stirring speed of 600 rpm, where the NaCl content actually decreases. Based on Fig. 7 (b), for the pre-treatment with chemical additions, it can be observed that double crystallisation at a crystallisation time of 100 min and a stirring speed of 600 rpm, a crystallisation time of 70 min and a stirring speed of 600 rpm, and a crystallisation time of 70 min and a stirring speed of 400 rpm increases the NaCl content. On the contrary, double crystallisation decreases the NaCl content for a crystallisation time of 100 min at a stirring speed of 400 rpm. In conclusion, in the pre-treatment without chemical additions, the number of crystallisations or double crystallisation tends to decrease the NaCl content. Conversely, in the pre-treatment with chemical additions, there is a tendency for longer crystallisation times to increase the NaCl content. This is inconsistent with the research by Ref. [11], which suggested that with the addition of chemicals, the NaCl content remains constant throughout the crystallisation period. This inconsistency may be because NaOH and Na_2_CO_3_ have already bound the impurities, so fewer impurities are attached to the crystals in the mother liquor. The inconsistency may also be due to incomplete filtration, which allows some Ca2+ and Mg2+ impurities to remain in the salt solution, resulting in non-constant NaCl content and increasing crystallisation time. The number of crystallisations or double crystallisation tends to increase the NaCl content. According to the research by Ref. [4], double crystallisation reduces impurity levels, leading to an increase in NaCl content. This is in line with the results of this study in the treatment with chemical additions. However, the results for the pre-treatment without chemical additions do not align with this. In the pre-treatment with chemical additions, there is a tendency for longer crystallisation times to increase the NaCl content.

#### 3.2.3 Magnesium content

The recrystallisation method has successfully reduced the magnesium impurities content in the salt from an initial level of 0.27 % or 2700 ppm to 0–497 ppm. Furthermore, the most effective treatment for reducing magnesium content is achieved in pharmaceutical salt production with the variable of chemical addition at a stirring speed of 600 rpm. This is because the obtained magnesium content is 0 ppm, which complies with the applicable pharmacopoeia standards [13], which require a magnesium content below 100 ppm.

The influence of crystallisation time on magnesium content is presented in the graph below.

**Fig. 8:**
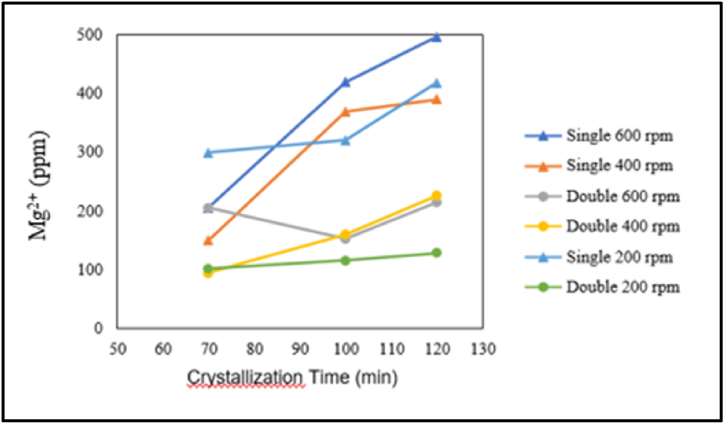
Relationship between crystallisation time and magnesium content.

Based on Fig. 8, in the case of single crystallisation treatment with stirring speeds of 600, 400, and 200 rpm, as well as double crystallisation treatment with stirring speeds of 400 and 200 rpm, there is an increase in magnesium content with increasing crystallisation time. Additionally, in the case of double crystallisation with a stirring speed of 600 rpm, the magnesium content at a crystallisation time of 100 min is lower than the magnesium content at crystallisation times of 70 and 120 min. From the graph above, in pharmaceutical salt production, there is an increase in magnesium content with increasing crystallisation time. This is because the demineralised water solvent evaporates more as the crystallisation time increases. Evaporating water produces salt and increases the impurity concentration research by Ref. [9]. The concentration of magnesium increases because magnesium ions (Mg^2+^) crystallise and become trapped in the salt crystals research by Ref. [11].

The influence of stirring speed on magnesium content is presented in the graph below.

**Figure: 9:**
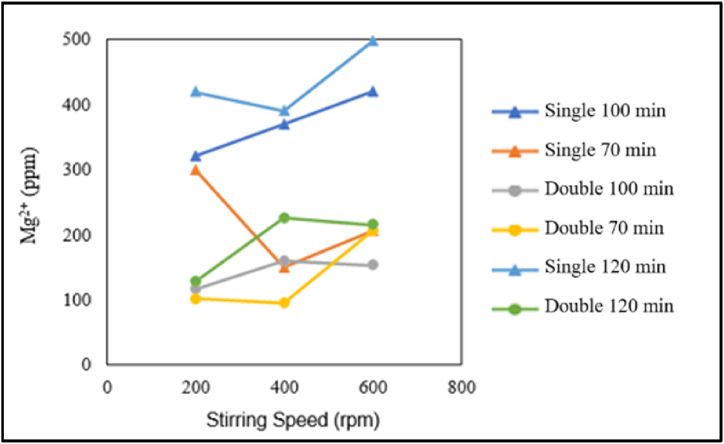
Relationship between stirring speed and magnesium content.

Based on Fig. 9, for pharmaceutical salt production with single crystallisation treatment at a crystallisation time of 120 min and 70 min, as well as double crystallisation treatment at a crystallisation time of 70 min, the lowest magnesium content is achieved at a stirring speed of 400 rpm compared to stirring speeds of 200 and 600 rpm. For pharmaceutical salt production with double crystallisation treatment at crystallisation times of 100 and 120 min, the magnesium content at a stirring speed of 400 rpm is higher than at stirring speeds of 200 and 600 rpm. However, for pharmaceutical salt production with single crystallisation treatment at a crystallisation time of 100 min, there is an increase in magnesium content from a stirring speed of 200–600 rpm. From the graph above, the relationship between magnesium content and stirring speed can be concluded. In pharmaceutical salt production variables, stirring speed does not have a consistent effect. With increasing stirring speed, there can be an increase or decrease in magnesium content. The increase in magnesium content with increasing stirring speed can be influenced by the evaporation of the solvent, which is demineralised water. This aligns with the literature that states that higher stirring rates lead to higher evaporation rates by Ref. [18]. Evaporating water produces salt and increases the impurity concentration research by Ref. [9]. The concentration of magnesium increases because magnesium ions (Mg^2+^) crystallise and become trapped in the salt crystals research by Ref. [11].

In contrast, a decrease is influenced by the porosity of NaCl crystals. Face-centred cubic NaCl solids have micropores that empty 26 % of the crystal volume research by Ref. [2]. These pores allow mother liquor to be absorbed into the NaCl crystals. Based on literature studies, increasing the stirring rate can reduce a crystal's agglomeration and porosity, resulting in denser crystals research by Astuti et al., 2016.

The influence of the amount of crystallisation on magnesium content is presented in the graph below.

**Fig. 10:**
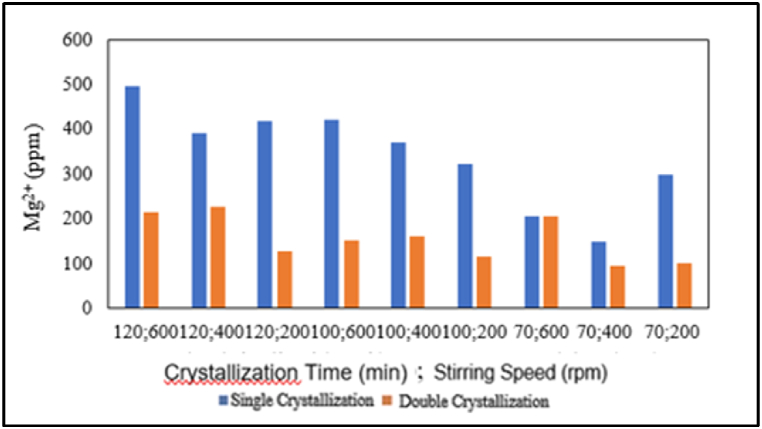
Relationship between the amount of crystallisation and magnesium content.

Based on Fig. 10, in the production of salt with a crystallisation time of 120 min at stirring speeds of 600, 400, and 200 rpm, the production of salt with a crystallisation time of 100 min at stirring speeds of 600, 400, and 200 rpm; and the production of salt with a crystallisation time of 70 min at stirring speeds of 400 and 200 rpm, there is a decrease in magnesium content with an increasing number of single or double crystallisations. In addition, the production of salt with a crystallisation time of 70 min at a stirring speed of 600 rpm has a constant magnesium content of 206 ppm, regardless of whether it undergoes single or double crystallisation treatment. From Fig. 10 above, it can be concluded that the decrease in magnesium content occurs with an increasing number of crystallisations (from single to double), which applies to pharmaceutical salt production variables with or without chemical additions. This is because, in the double crystallisation treatment, there are two crystallisation processes, which cause more NaCl and impurities, especially magnesium (Mg^2+^), to be dissolved in the mother liquor compared to a single crystallisation research by Ref. [4].

The influence of the addition of NaOH on magnesium content is presented in the graph below.

**Fig. 11:**
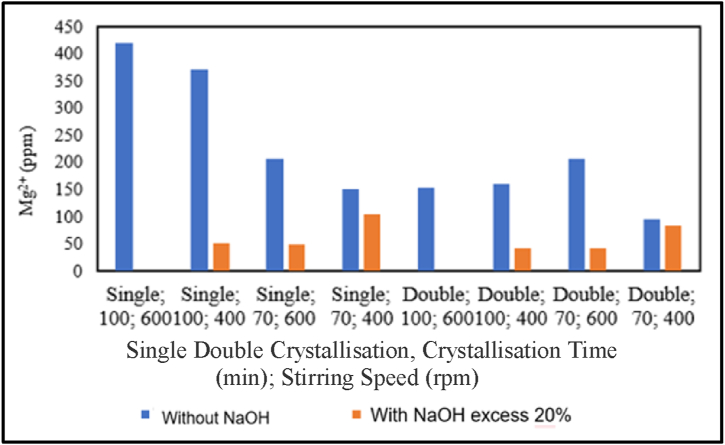
Relationship between the addition of NaOH and magnesium content.

Based on Fig. 11, in pharmaceutical salt production with single crystallisation treatment at a crystallisation time of 100 min and stirring speeds of 600 and 400 rpm, single crystallisation at a crystallisation time of 70 min at stirring speeds of 600 rpm and 400 rpm, double crystallisation treatment at a crystallisation time of 100 min at a stirring speed of 600 rpm, and double crystallisation treatment at a crystallisation time of 70 min at a stirring speed of 600 rpm, there is a decrease in magnesium content with the addition of NaOH at an excess of 20 %. In addition, magnesium content increases in the double crystallisation treatment with a crystallisation time of 100 min and 70 min at a stirring speed of 400 rpm. From Fig. 11, there is a decrease in magnesium content with increasing crystallisation time. This is inconsistent with the literature, which states that binding magnesium (Mg^2+^) by NaOH at the same stirring time during pre-treatment eliminates a constant magnesium content in the salt, which is 0 ppm. The addition of a precipitating agent in the form of sodium hydroxide (NaOH) causes magnesium (Mg^2+^) to form magnesium hydroxide (Mg(OH)_2_) research by Ref. [19]. Therefore, crystallisation time does not affect magnesium content (Mg^2+^). The filtration procedure can influence the inconsistency in magnesium content, which allows some Mg(OH) _2_ to pass into the filtrate, which will then be crystallised. Fig. 11 shows that there is a decrease in magnesium content with increasing stirring speed. This is influenced by the greater effect of the precipitating agent compared to the stirring speed. The precipitating agent, sodium hydroxide (NaOH), causes magnesium (Mg^2+^) to form magnesium hydroxide (Mg(OH)_2_) research by Ref. [19].

Furthermore, Fig. 11 shows a decrease in magnesium content with increasing single or double crystallisations. However, in pharmaceutical salt production with a crystallisation time of 100 min at a stirring speed of 600 rpm, the magnesium content reached 0 ppm when undergoing single or double crystallisation treatment. This is influenced by the greater effect of the precipitating agent compared to the stirring speed. The precipitating agent, sodium hydroxide (NaOH), causes magnesium (Mg^2+^) to form magnesium hydroxide (Mg(OH)_2_) research by Ref. [19]. From the graph, it can be concluded that pharmaceutical salt production with the variable of pre-treatment involving the addition of the precipitating agent NaOH leads to a decrease in magnesium content. This is due to the binding reaction of magnesium (Mg^2+^) by the sodium hydroxide (NaOH) solution, which forms a solid product of magnesium hydroxide (Mg(OH)_2_) that is insoluble in water and tends to precipitate research by Ref. [19]. The reactions of NaOH are as follows:

(1) MgCl_2 (aq)_ + 2NaOH _(aq)_ → Mg(OH)_2 (s)_ + 2NaCl _(aq)_ … … … … … … … … ….

(2) MgSO_4 (aq)_ + 2NaOH _(aq)_ → Mg(OH)_2 (s)_ + Na_2_SO_4 (aq)_ … … … … … … … … ….

Mg, which carries a double positive charge, exhibits strong attraction to OH^−^ ion. Therefore, the bond formed during the coagulation reaction of NaOH with impurities is ionic. The solubility of Mg(OH)_2_ is lower than NaCl. These ions will react and form a solid compound with lower solubility, following the principle known as the solubility product law, where certain compounds will precipitate when their solubility in a solution is exceeded. Based on this principle, Mg^2+^ will react with OH^−^, forming a solid precipitate, Mg(OH)_2_, which is then separated by filtration from the solution.

#### 3.2.4 Calcium content

Regarding the salt content test results, it can be concluded that the recrystallisation method has successfully reduced the impurity level of calcium in the salt from its initial value of 0.24 % or 2400 ppm to 41.2–213 ppm. Furthermore, the most effective treatment for reducing calcium content was achieved by producing pharmaceutical salt with variable chemical additives at a stirring speed of 600 rpm. This is because the obtained calcium content is 41.2 ppm, which complies with the applicable pharmacopoeia standards of less than 100 ppm [13]. The following discusses the relationship between calcium content and the existing variables.

The influence of crystallisation time on calcium content is presented in the graph below.

**Fig. 12:**
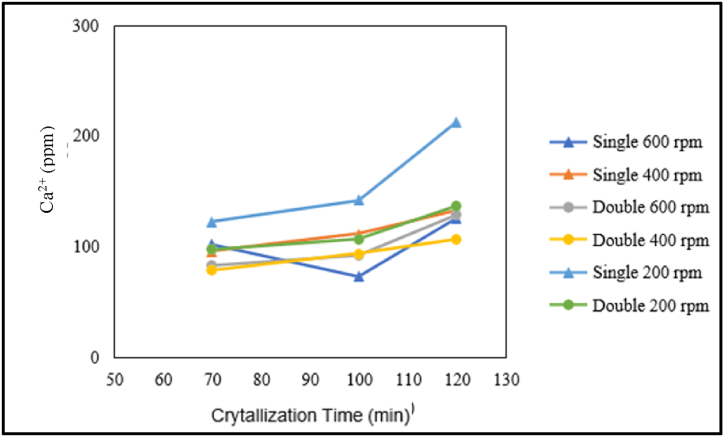
Graph of the relationship between crystallisation time and calcium content.

Based on Fig. 12, in the case of single crystallisation treatment at stirring speeds of 600 and 400 rpm and double crystallisation treatment at stirring speeds of 600, 400, and 200 rpm, there is an increase in calcium content with increasing crystallisation time. In addition, in the single crystallisation treatment at a stirring speed of 200 rpm, the calcium content at a crystallisation time of 100 min is lower than that at crystallisation times of 70 and 120 min. From the graph above, in the production of pharmaceutical salt, there is an increase in calcium content with increasing crystallisation time. This is because the solvent in demineralised water evaporates more as the crystallisation time increases. Evaporating water not only results in the production of salt but is also accompanied by an increase in the concentration of impurities research by Ref. [9]. The concentration of calcium increases due to the crystallisation of calcium (Ca^2+^), with more of it getting trapped within the salt crystals [14].

The influence of stirring speed on calcium content is presented in the graph below.

**Fig. 13:**
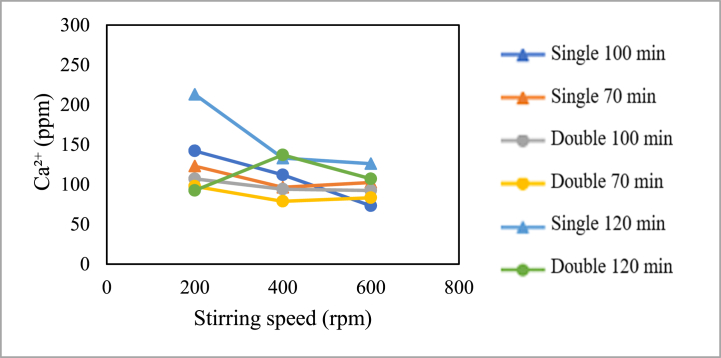
Graph of the relationship between crystallisation time and calcium content.

Fig. 13 shows the relationship between stirring speed and calcium (Ca^2+^) content in the production of pharmaceutical salt with variable pre-treatment without the addition of chemicals. Based on the graph, several correlations can be observed as follows. For the production of pharmaceutical salt with single crystallisation treatment with a crystallisation time of 70 min and double crystallisation treatment with crystallisation times of 100 and 70 min, the calcium (Ca^2+^) content is lowest at a stirring speed of 400 rpm compared to stirring speeds of 200 and 600 rpm at the same crystallisation time. For the production of pharmaceutical salt with double crystallisation treatment with crystallisation times of 100 and 120 min, the calcium content decreases as the crystallisation time increases. However, for the production of pharmaceutical salt with double crystallisation treatment with a crystallisation time of 120 min, there is an increase in calcium content at 400 rpm compared to stirring speeds from 200 to 600 rpm.

From the above graph, it can be concluded that there is a relationship between calcium content and stirring speed. In the production of pharmaceutical salt without adding chemicals as a variable, the stirring speed does not consistently affect the calcium content. With an increase in stirring speed, there can be an increase or decrease in magnesium content. The evaporation of solvents, such as demineralised water, can influence the increase in magnesium content with increasing stirring speed. This is consistent with literature studies stating that the higher the stirring rate, the higher the evaporation rate [17]. Salt is produced by evaporating water, which increases impurity concentration [2]. The calcium concentration is caused by calcium (Ca^2+^) that also crystallises, and more of it becomes trapped in the salt crystals [14].

Meanwhile, the decrease is influenced by the porosity of NaCl crystals. NaCl solids in face-centred cubic crystals have micropores that empty 26 % of the crystal volume [20]. These pores allow the mother liquor to be absorbed into the NaCl crystals. Based on literature studies, it is stated that increasing stirring speed can reduce the agglomeration and porosity of a crystal, resulting in denser crystals [15].

The influence of the amount of crystallisation on calcium content is presented in the graph below.

**Fig. 14:**
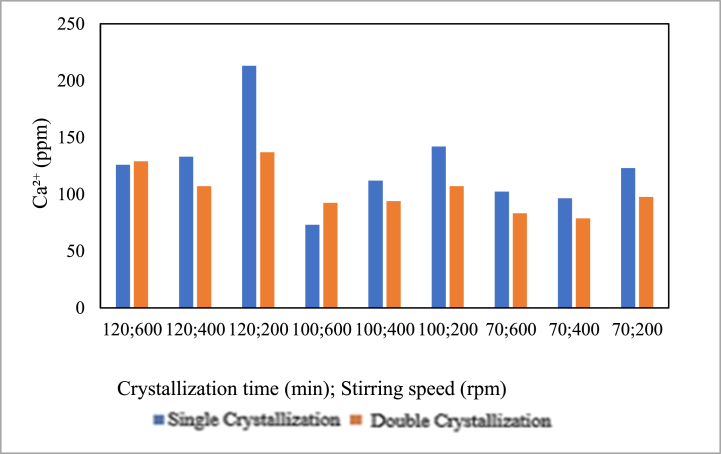
Relationship between the amount of crystallisation and calcium content.

Based on Fig. 14 for the pre-treatment without the addition of chemicals, it can be observed that the double crystallisation treatment for 120 min at 400 rpm, 100 min at 600 rpm, 100 min at 400 rpm, 70 min at 400 rpm, and 70 min at 400 rpm managed to decrease the calcium (Ca^2+^) content. However, at 120 min and 600 rpm with the double crystallisation treatment, there was an increase in calcium (Ca^2+^) content. The abundance of crystallisation or double crystallisation tends to decrease the calcium (Ca^2+^) content. This is in line with the study by Geotzfried and Kondorosy, 2018, which states that double crystallisation can reduce impurity levels, including calcium (Ca^2+^), in salt purification. The decrease in Ca^2+^ values is not very significant due to the long double crystal time, causing a lot of solvent evaporation and making the calcium (Ca^2+^) concentration denser and sticking to the salt crystals.

The influence of the addition of Na_2_CO_3_ on magnesium content is presented in the graph below.

**Fig. 15:**
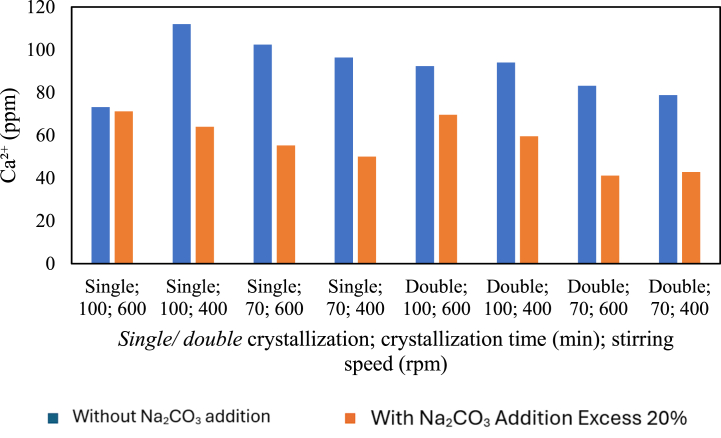
Relationship between the addition of Na_2_CO_3_ and calcium content.

Fig. 15 shows the relationship between adding the precipitating agent Na_2_CO_3_ in the pre-treatment of pharmaceutical salt production and the calcium content. Pre-treatment with chemical additions can be seen where the double crystallisation treatment for 100 min at 600 rpm, 100 at 400 rpm, 70 at 400 rpm, and 70 at 400 rpm decreased the calcium (Ca^2+^) content. In the variables with chemical additions, there was an increase in calcium content with increasing stirring speed. This contradicts the literature study, which states that binding calcium (Ca^2+^) by Na2CO3 at the same stirring speed in pre-treatment (i.e., 400 rpm) results in a constant calcium content in the salt, which is 0 ppm. Therefore, stirring speed does not affect the calcium (Ca^2+^) content. The inconsistency in calcium content can be influenced by the filtration procedure, which causes some CaCO_3_ to still pass through the filtrate at certain variable stirring speeds, which will then be crystallised.

Additionally, from Fig. 15, in the production of pharmaceutical salt with single crystallisation treatment for 100 min at 400 rpm, single crystallisation for 70 min at 600 rpm and 400 rpm, double crystallisation for 100 min at 600 and 400 rpm, and double crystallisation for 70 min at 600 and 400 rpm, there was a decrease in calcium content with the addition of Na_2_CO_3_ at an excess of 20 %.

Furthermore, a non-significant decrease in calcium content occurred in the single crystallisation treatment for 100 min at 600 rpm. From the graph, it can be concluded that the production of pharmaceutical salt with the variable of pre-treatment addition of a precipitating agent such as Na_2_CO_3_ causes a decrease in calcium content. This is due to the reaction of binding magnesium (Ca^2+^) by sodium carbonate solution (Na_2_CO_3_) which forms solid calcium carbonate (CaCO_3_) products that are insoluble in water and tend to precipitate [7]. When Na_2_CO_3_ is added, impurities such as Ca^2+^ should bind to Na_2_CO_3_, resulting in constant calcium content in the presence of chemical additives, regardless of whether crystallisation time increases or decreases research by Ref. [11]. The inconsistency observed is likely due to imperfect filtration or the presence of Ca^2+^ that passes through during the filtration process. The reaction is as follows:

(4) CaCl_2 (aq)_ + Na_2_CO_3 (aq)_ → CaCO_3 (s)_ + 2NaCl_(aq)_ … … … … … … … ….… … … … ….

…(5) CaSO_4 (aq)_ + Na_2_CO_3 (aq)_ → CaCO_3(s)_ + Na_2_SO_4(aq)_ … … … … … … … ….… … … …

When Na_2_CO_3_ is mixed with the solution of processed salt, it binds to Ca^2+^ ions present in salts such as CaCl_2_ and CaSO_4_, forming CaCO_3_ as a solid precipitate that can be separated through filtration. This occurs due to ion exchange between Ca^2+^ from CaSO_4_ and CaCl_2_ with CO_3_^2−^ from Na_2_CO_3_, which is soluble in water. This is by the solubility product law, as the solubility of CaCO_3_ is lower than that of NaCl.

#### 3.2.5 Sulfate level

It can be concluded that the crystallisation method has reduced the sulfate salt content from 1.88 % or 18,800 ppm to 316–719 ppm. However, the sulfate content obtained still needs to comply with the pharmacopoeia standards applicable to pharmaceutical salts, which require a sulfate content below 200 ppm [13]. This is because the raw material has a very high sulfate content, necessitating further treatment such as washing or ion exchange to achieve the 200 ppm sulfate standard. Nevertheless, the influence of variables on sulfate content can be inferred. As a temporary solution to reduce sulfate content that does not meet the standard, additional runs were conducted in which the double crystallisation time was reduced from half of the initial first crystallisation time to one-third of the first. The result of this treatment successfully yielded a sulfate content of 64 ppm, which meets the pharmacopoeia standard of sulfate (SO_4_^2−^) content being less than 200 ppm. However, the drawback is that reducing the crystallisation time resulted in a lower mass and yield of pharmaceutical salt.

The influence of crystallisation time on sulfate content is presented in the graphs below.

**Fig. 16:**
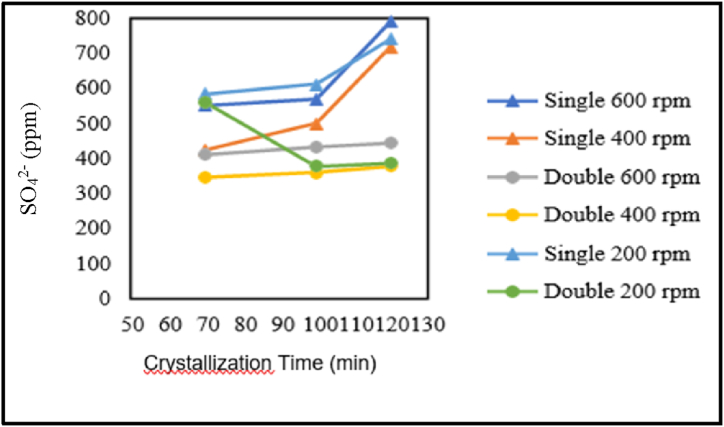
Of the relationship between crystallisation time and sulfate content.

Based on Fig. 16 in the production of pharmaceutical salt with single crystallisation treatment at stirring speeds of 600 rpm, 400 rpm, and 200 rpm, as well as double crystallisation treatment at stirring speeds of 600 and 400 rpm, there is an increase in sulfate content with increasing crystallisation time. In addition, for the production of pharmaceutical salt with single crystallisation treatment at a stirring speed of 600 rpm, a crystallisation time of 100 min results in the lowest magnesium content compared to crystallisation times of 70 and 120 min. Both graphs show that sulfate content increases with increasing crystallisation time, whether with or without adding chemical substances. This is because the solvent, demineralised water, evaporates more as the crystallisation time increases. On the other hand, there is a concentration of sulfate (SO_4_^2−^), and sulfate (SO_4_^2−^) crystals become increasingly trapped within the salt crystals research by Ref. [11].

The influence of stirring speed on sulfate content is presented in the graph below.

**Fig. 17:**
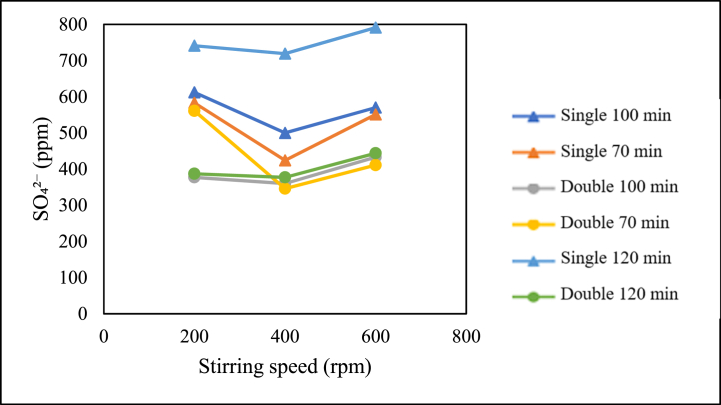
Graph of the relationship between stirring speed and sulfate content.

Fig. 17 indicates the relationship between stirring speed and sulfate content in the production of pharmaceutical salt with variable pre-treatment without the addition of chemicals. Based on the graph, for the production of pharmaceutical salt with single/double crystallisation treatment for 70, 100, and 120 min, the lowest sulfate content was obtained at a stirring speed of 400 rpm compared to stirring speeds of 200 and 600 rpm.

From the graph above, it can be concluded that there is a relationship between sulfate content and stirring speed. In the production of pharmaceutical salt with variables without the addition of chemicals, the speed does not consistently affect the sulfate content. However, for each variable of single/double crystallisation treatment using stirring times of 70, 100, and 120 min, the lowest sulfate content was obtained using a speed of 400 rpm compared to 200 and 600 rpm. The impurity content can be influenced by solvent evaporation and crystal porosity. The evaporation of solvents, such as demineralised water, influences the increase in sulfate content with increasing stirring speed. This is consistent with literature studies stating that the higher the stirring rate, the higher the evaporation rate [17]. By evaporating water, salt is produced and an increase in impurity concentration [19]. The concentration of impurities is caused by sulfate (SO_4_^2−^), which also crystallises, and more of it becomes trapped in the salt crystals [14].

Meanwhile, the decrease in impurity content is influenced by the porosity of NaCl crystals. NaCl solids in the form of face-centred cubic crystals have micropores that empty 26 % of the crystal volume [20]. These pores allow the mother liquor to be absorbed into the NaCl crystals. Based on literature studies, it is stated that increasing stirring speed can reduce the agglomeration and porosity of a crystal, resulting in denser crystals [15].

The influence of the amount of crystallisation on sulfate content is presented in the graph below.

**Fig. 18:**
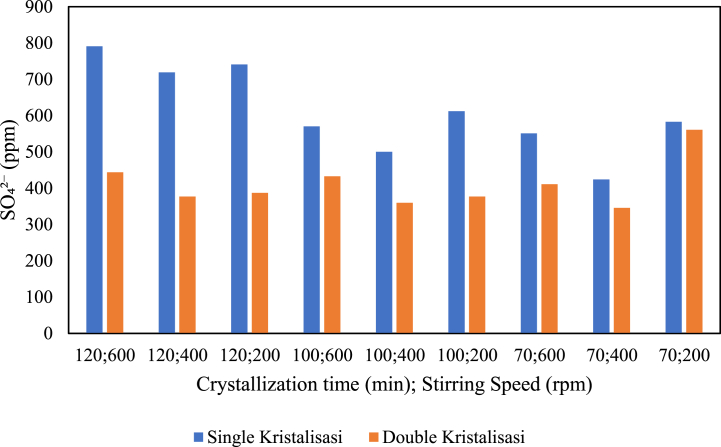
Relationship between the amount of crystallisation and sulfate content.

Fig. 18 indicates the relationship between the number of crystallisations and sulfate content in the production of pharmaceutical salt with variable pre-treatment without the addition of chemicals. Based on the graph, an increase in the number of crystallisations leads to a decrease in sulfate content in pharmaceutical salt. This applies to the production of salt with a crystallisation time of 100 min at stirring speeds of 600 and 400 rpm and the production of salt with a crystallisation time of 70 min at stirring speeds of 600 and 400 rpm. From Fig. 18, it can be concluded that the decrease in sulfate content occurs with an increasing number of crystallisations. Based on literature studies, additional treatments are required to obtain pharmaceutical salt with low sulfate content, such as double crystallisation, to reduce sulfate from the brine. The salt from crystallisation is dissolved into water, and the crystallisation process continues.

The influence of the addition of Na_2_CO_3_ on magnesium content is presented in the graph below.

**Fig. 19:**
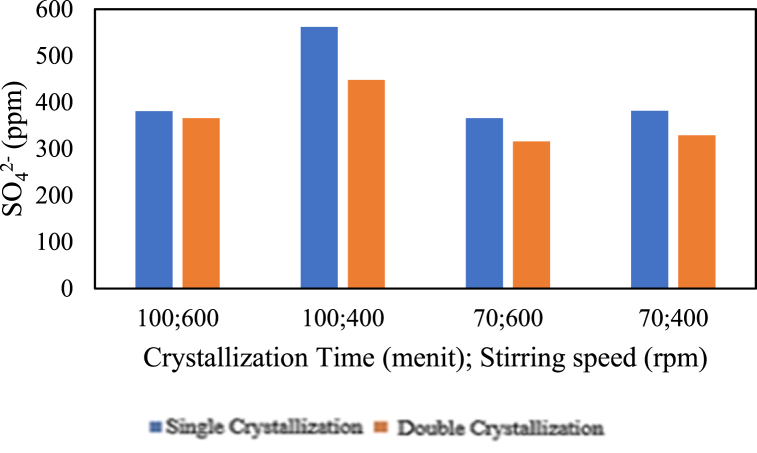
Relationship between the addition of Na_2_CO_3_ and sulfate content.

Based on Fig. 19, it can be observed that for the production of pharmaceutical salt with single crystallisation treatment at stirring speeds of 600 rpm and 400 rpm, as well as double crystallisation treatment at a stirring speed of 400 rpm, there is an increase in sulfate content with increasing crystallisation time. Additionally, sulfate content decreases with increasing crystallisation time in the production of pharmaceutical salt with double crystallisation treatment at a stirring speed of 400 rpm. Next is the relationship between stirring speed and sulfate content in the production of pharmaceutical salt with variable pre-treatment and chemical additions; it can be seen that there is a decrease in sulfate content with increasing stirring speed. This applies to the production of pharmaceutical salt with single crystallisation treatment for 70 and 100 min and double crystallisation treatment for 70 and 100 min. After chemical additions, there is a tiny decrease in sulfate content with increasing stirring speed, which shows that chemical addition did not significantly affect SO_4_^2−^ reduction. This aligns with previous research [17] that the higher the stirring rate, the higher the evaporation rate. High evaporation leads to the concentration of impurities in the salt, causing impurities to adhere more to the salt crystals [14]. Therefore, stirring speed does not significantly affect sulfate (SO_4_^2−^) impurity levels.

Furthermore, Fig. 19 shows that an increase in the number of crystallisations leads to decreased sulfate content in pharmaceutical salt. This applies to the production of salt with a crystallisation time of 100 min at stirring speeds of 600 and 400 rpm and the production of salt with a crystallisation time of 70 min at stirring speeds of 600 and 400 rpm. The reduction in sulfate content with chemical addition treatment has not been able to meet pharmacopoeia standards, which state a maximum of 200 ppm, due to the high sulfate content in the processed salt raw material of 11800 ppm [4]. According to Ref. [4], a reduction in sulfate content can be achieved up to 86 ppm due to the sulfate content in the raw material, which is a brine with a sulfate content of 3 g/L or 3000 ppm, which is lower than the sulfate content in the processed salt.

#### 3.2.6 Potassium content

Recrystallisation can reduce the potassium content, initially 0.22 % or 2200 ppm in the processed salt raw material. After the recrystallisation process with various variables, the potassium content in the salt falls within the range of 0.033 %–0.0391 %, approximately 330–391 ppm. According to pharmaceutical salt standards, the potassium content (K^+^) must be < 500 ppm [13].

The influence of crystallisation time on potassium content is presented in the graphs below.

**Fig. 20:**
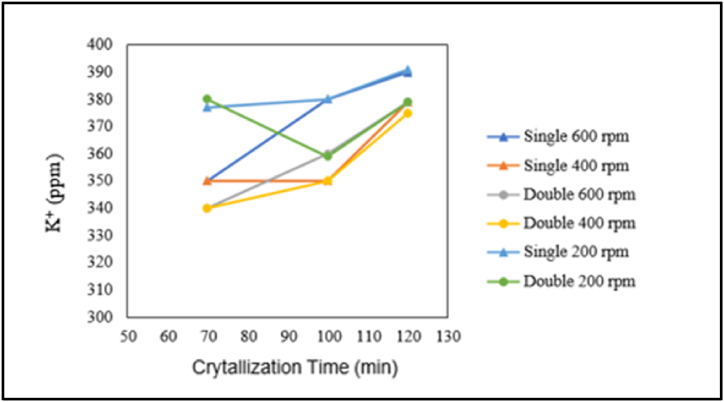
Graph of the relationship between crystallisation time and potassium content.

Based on Fig. 20, it can be observed that both single crystallisation and double crystallisation at stirring speeds of 200, 400, or 600 rpm result in an increase in potassium content with increasing crystallisation time. In both pre-treatment with and without the addition of chemicals, potassium content (K^+^) tends to increase with increasing crystallisation time. This is consistent with research by Ref. [14], which indicates that as time passes, more solvent evaporates, leading to an increase in the concentration of impurities in the solution, including potassium (K^+^), resulting in more potassium being trapped or adhering to salt crystals.

The influence of stirring speed on sulfate content is presented in the graph below.

**Fig. 21:**
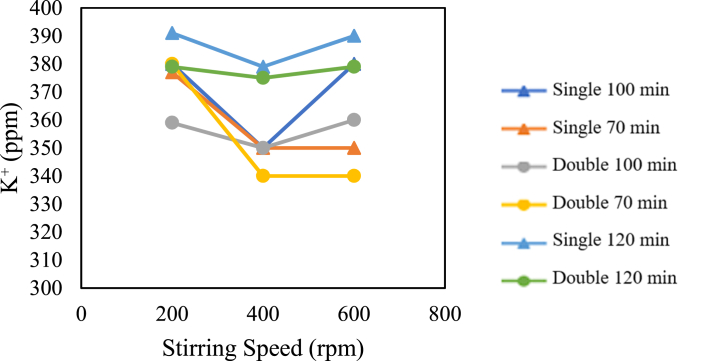
Graph of the relationship between stirring speed and sulfate content.

In Fig. 21, for the pre-treatment without the addition of chemicals, both for single and double crystallisation treatments, whether for a crystallisation time of 70 min, 100 min, or 120 min, at a stirring speed of 200 rpm, the potassium content is higher than the potassium content at a stirring speed of 400 rpm. It can also be seen that at a stirring speed of 400 rpm, the potassium content is lower than that at a stirring speed of 600 rpm. This is true except for the double and single crystallisation treatments with a crystallisation time of 70 min, where the potassium content at stirring speeds of 400 rpm and 600 rpm is the same.

In general, the most optimal stirring speed or the lowest potassium (K^+^) content is at 400 rpm. This occurs because as the stirring speed increases, more solute evaporates, resulting in a more concentrated potassium solution. The increasing potassium concentration causes more potassium to be trapped or adhere to the salt crystals. This is why the potassium content tends to be higher at a stirring speed of 600 rpm. The research supports this [17], stating that the higher the stirring rate, the higher the evaporation rate. This statement is also supported by the research [14] stating that as more solvent is evaporated, the concentration of impurities such as potassium (K^+^) becomes more concentrated, leading to more potassium (K^+^) being trapped in the salt crystals.

On the other hand, as the stirring speed increases, the porosity of the salt crystals decreases, so the crystals do not absorb as much mother liquor-containing impurities, leading to a decrease in NaCl content. This aligns with the research [15] stating that higher stirring speeds result in smaller porosity due to denser and stronger particle bonds. The research [16] also supports this, stating that higher porosity values result in higher fluid absorption or more fluid adhering to the crystals. This is why, at a stirring speed of 200 rpm, the potassium content is high; the high potassium content is due to its high porosity, which triggers the crystals to absorb more solution, leading to more potassium adhering to the crystals. The stirring speed of 400 rpm results in the lowest potassium content because, at this speed, the porosity is not too high, so there is not much solution adhering to the salt crystals, and the concentration of the solution adhering is not too high because the evaporation rate is adequate.

The influence of the amount of crystallisation on potassium content is presented in the graph below.

**Fig. 22:**
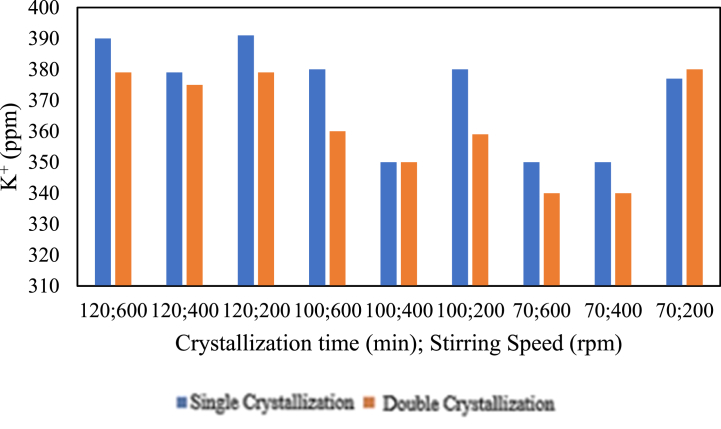
Relationship Between the Amount of Crystallisation and potassium Content.

Based on Fig. 22, it can be observed that the double crystallisation treatment for 120 min at all stirring speeds, 100 min at stirring speeds of 600 and 200 rpm, and 70 min at stirring speeds of 600 and 400 rpm resulted in a decrease in potassium (K^+^) content. On the other hand, for a crystallisation time of 100 min at a stirring speed of 400 rpm, the double crystallisation treatment did not change the potassium (K^+^) content; it remained the same. Additionally, the potassium content increased with the double crystallisation process for a crystallisation time of 70 min at a stirring speed of 200 rpm. The abundance of crystallisation or double crystallisation, both before and after chemical addition, tends to decrease the potassium (K^+^) content. This is in line with the study by Ref. [4], which states that double crystallisation can reduce impurity levels, including potassium (K^+^), in salt purification.

The influence of chemical addition on potassium content is presented in the graph below.

**Fig. 23:**
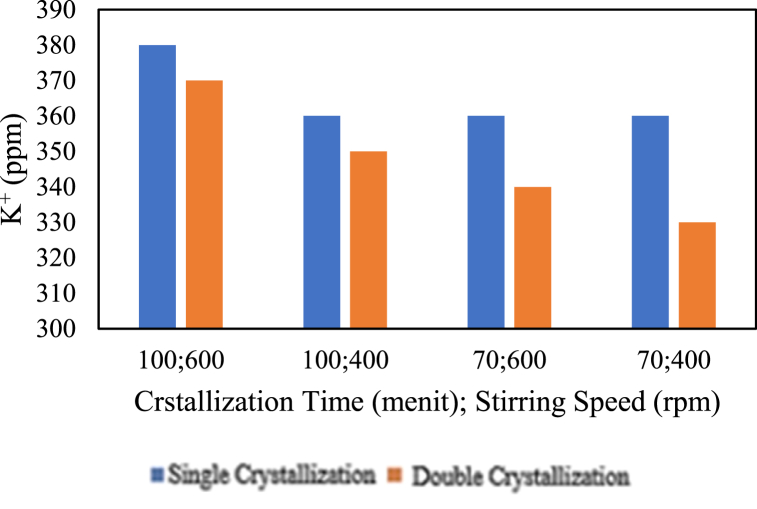
Relationship between chemical addition and potassium content.

Based on Fig. 23, for the pre-treatment with the addition of chemicals, it can be seen that for single crystallisation at a stirring speed of 400 rpm, the potassium content does not change with changes in crystallisation time. In the case of single crystallisation at a stirring speed of 400 rpm, changes in crystallisation time do not affect the potassium content (K^+^). However, for both double crystallisation at stirring speeds of 600 rpm and 400 rpm and single crystallisation at 600 rpm, the potassium content (K^+^) increases with increasing crystallisation time. In Fig. 23, for the pre-treatment with chemical additions, it can be observed that increasing the stirring speed will increase the potassium (K^+^) content in the variables with double crystallisation for 70 min or 100 min of crystallisation time. However, for the variables with single crystallisation for 70 min or 100 min, increasing the stirring speed does not affect the potassium (K^+^) content; it remains the same. For the pre-treatment with chemical additions, it can also be seen that the double crystallisation treatment for 100 min at 600 rpm, 100 min at 400 rpm, 70 min at 400 rpm, and 70 min at 400 rpm decreased the potassium (K^+^) content. After adding chemicals, the obtained salt has no significant decrease in potassium content. However, the potassium content is lower than without adding chemicals, and these values are still within pharmaceutical standards of <500 ppm.

### 3.3 FTIR test result

**Fig. 24:**
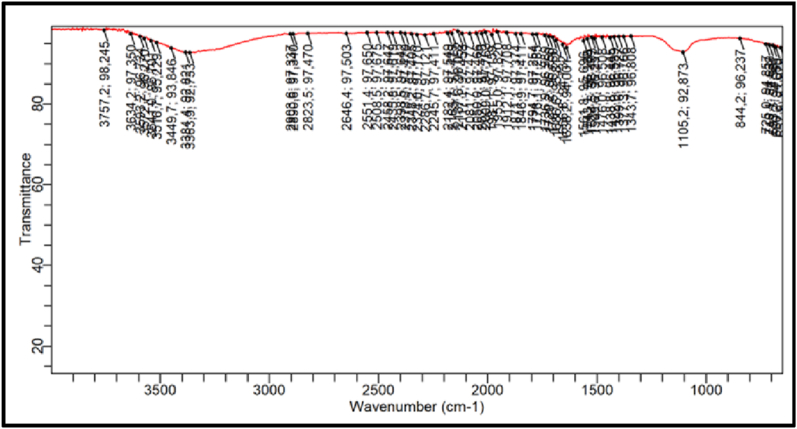
FTIR Result Of pharmaceutical salt.

According to Fig. 24, the results of the FTIR spectrum analysis reveal a sharp peak at the wavelength of 3383.92 cm-1 with broad stretching vibration, characteristic of the asymmetric OH group. This is consistent with the literature from previous studies [12,21] on magnesium hydroxide precipitates.

### 3.4 SEM test result

**Fig. 25:**
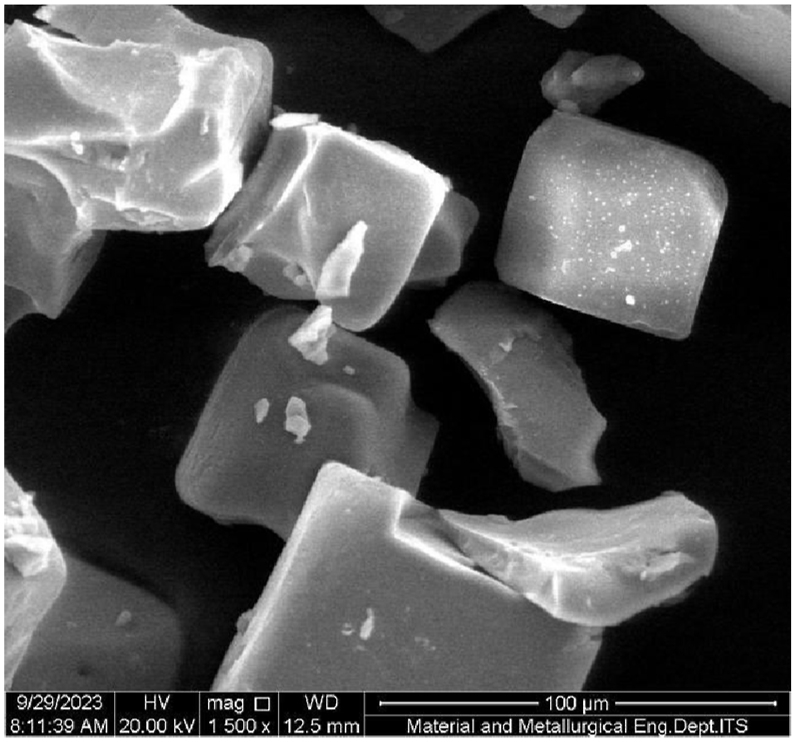
Mikrograf SEM pharmaceutical salt.

According to Fig. 25, the result of scanning electron microscopy (SEM) morphology of pharmaceutical salt assumes a cubic crystal structure, which corresponds to the crystalline form of NaCl. The NaCl crystal exhibits a cubic crystal shape with a space group designated as Fm3m and a crystal lattice length measuring 5.620 Å. The uneven surface of the cube is identified as impurities adhering to the crystal during the crystal seed enlargement process research by Ref. [22].

### 3.5 XRD test result

**Fig. 26:**
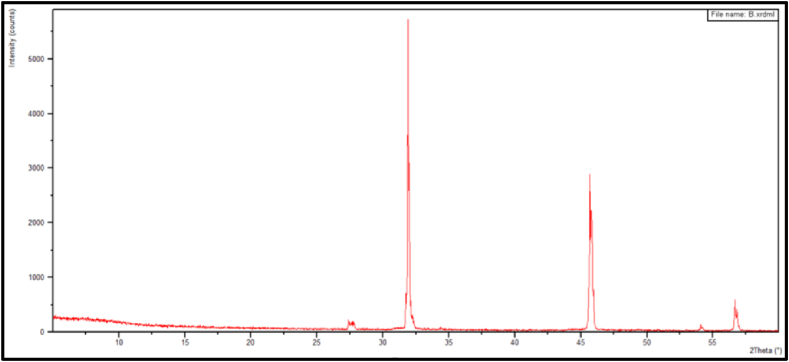
XRD Pattern of pharmaceutical salt.

Based on the results of the XRD pattern analysis in Fig. 26, several main peaks are observed. The prominent peaks, deal values, and relative intensities from the X-ray diffraction of the pharmaceutical salt indicate that the significant peaks correspond to those of the NaCl crystal. Therefore, it can be concluded that the pharmaceutical salt predominantly consists of NaCl compound, with impurities including Mg, Ca, SO_4_, and K.

## 4 Conclusion

The best condition for the studied variables in pharmaceutical salt production is double crystallisation with the addition of a 20 % excess of chemicals at a crystallisation time of 100 min and a stirring speed of 600 rpm. The salt composition included NaCl at 99.87 %, Mg^2+^ at 0 ppm, Ca^2+^ at 69.6 ppm, SO_4_^2−^ at 366 ppm, K^+^ at 370 ppm, and a water content of 0.166, yielded at 15 %. The yield obtained from double crystallisation was lower than single after chemical addition to obtain a higher quality of pharmaceutical salt by increasing NaCl content and reducing impurities content (Water, Mg^2+^, Ca^2+^, K+, SO_4_^2−^). However, the SO_4_^2−^ content did not meet pharmacopoeia standards in this research. The SO_4_^2−^ content can meet pharmacopoeia standards if the second crystallisation time is reduced to 1/3 of the first crystallisation time. Recrystallisation alone is not practical in producing pharmaceutical salt from processed salt. As the crystallisation time increases, the yield increases while the content decreases. The effect of crystallisation time on impurity levels (Mg^2+^, Ca^2+^, SO_4_^2−^, and K^+^) is directly proportional, with the lowest impurity levels on average obtained at a crystallisation time of 70 min. Higher stirring speeds result in smaller yields. The highest NaCl content and the lowest impurity levels (Mg^2+^, Ca^2+^, SO_4_^2−^, and K^+^) are typically obtained at a stirring speed of 400 rpm. Increasing the number of crystallisations (double crystallisation) leads to lower yields and impurity levels while the NaCl content increases. The addition of precipitating agents reduces the yield. Meanwhile, adding precipitating agents (NaOH and Na_2_CO_3_) increases the NaCl content and reduces the impurity levels (Mg^2+^ and Ca^2+^).

## CRediT authorship contribution statement

**T. Widjaja:** Funding acquisition, Formal analysis, Data curation, Conceptualization. **A. Altway:** Data curation. **S. Nurkhamidah:** Methodology, Data curation. **Y. Rahmawati:** Methodology. **W. Meka:** Validation, Methodology. **A. Alifatul:** Writing – review & editing, Writing – original draft, Project administration, Methodology, Formal analysis, Data curation, Conceptualization. **D. Hartanto:** Data curation, Conceptualization. **R. Sari:** Methodology, Conceptualization.

## Declaration of competing interest

The authors declare that they have no known competing financial interests or personal relationships that could have appeared to influence the work reported in this paper.
